# Autophagy in Parkinson’s Disease

**DOI:** 10.3390/biom13101435

**Published:** 2023-09-22

**Authors:** Lior Nechushtai, Dan Frenkel, Ronit Pinkas-Kramarski

**Affiliations:** Department of Neurobiology, School of Neurobiology, Biochemistry and Biophysics, Tel-Aviv University, Ramat-Aviv, Tel Aviv 69978, Israel; lior.nech@gmail.com (L.N.); dfrenkel@tauex.tau.ac.il (D.F.)

**Keywords:** apolipoprotein E4 (apoE4), autophagy, endocytosis, lysosomal degradation, Parkinson’s disease (PD), synuclein α, endocytosis

## Abstract

Parkinson’s disease (PD) is a devastating disease associated with accumulation of α-synuclein (α-Syn) within dopaminergic neurons, leading to neuronal death. PD is characterized by both motor and non-motor clinical symptoms. Several studies indicate that autophagy, an important intracellular degradation pathway, may be involved in different neurodegenerative diseases including PD. The autophagic process mediates the degradation of protein aggregates, damaged and unneeded proteins, and organelles, allowing their clearance, and thereby maintaining cell homeostasis. Impaired autophagy may cause the accumulation of abnormal proteins. Incomplete or impaired autophagy may explain the neurotoxic accumulation of protein aggregates in several neurodegenerative diseases including PD. Indeed, studies have suggested the contribution of impaired autophagy to α-Syn accumulation, the death of dopaminergic neurons, and neuroinflammation. In this review, we summarize the recent literature on the involvement of autophagy in PD pathogenesis.

## 1. Introduction

**Figure 1:**
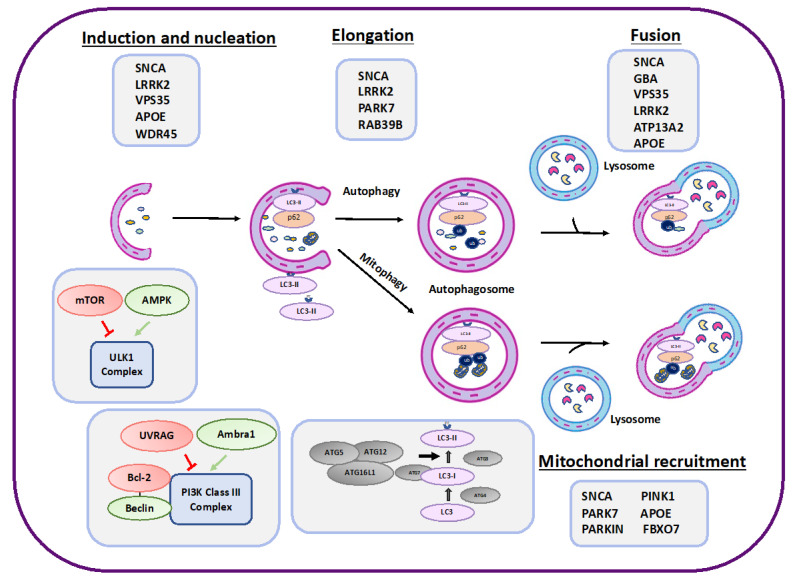
A schematic presentation of the various autophagy steps and proteins involved in autophagy regulation (lower part of the scheme. The red color represent negative regulators and the green color refers to positive regulators). The various proteins that affect various stages of autophagy and may also be involved in PD are listed in the gray boxes of the scheme (see also Table 1).

Parkinson’s disease (PD) is characterized by motor symptoms including bradykinesia, resting tremor and rigidity, and non-motor symptoms such as cognitive impairment and depression [1,2,3]. A major problem in the detection of PD is that diagnosis occurs with the onset of motor symptoms which are observed when the dopaminergic neurons are already dead, and the prodromal phase includes non-motor symptoms which are nonspecific. Therefore, it is important to identify molecular mechanisms and biomarkers or other diagnostic tools for early detection and maybe prevention [2]. Among the hallmarks of the disease is the accumulation of α-Syn and the death of dopaminergic neurons. Parkinson’s disease has genetic and non-genetic risk factors. Several environmental risk factors for PD have been suggested, such as exposure to pesticides, and traumatic brain injury [4]. Only 5–10% of PD cases are genetic and known to have monogenetic forms [5]. Several genes have been associated with increase in onset of PD (see Table 1). It was previously shown that point mutation in the synuclein alpha (*SNCA*) gene which encodes for α-Syn, leads to familial PD [2]. Moreover, duplication or triplication of the *SNCA* gene may lead to PD, although these are more rare cases [2]. Other mutations in the SNCA gene typical of familial PD include A53T (Ala53Thr), A30P (Ala30Pro), and E46K (Glu46Lys) [6]. Additional genes associated with familial PD which are discussed in Section 3 and Section 4, and presented in Figure 1 and Table 1, are *PARK7*, *LRRK2*, *APOE*, *PINK1*, *PRKN*, *GBA*, *VPS35*, *RAB39B*, *ATP13A2* (*PARK9*), *WDR45*, and *FBXO7* [7]. It was previously suggested that autophagy, a self-degradative process which is important for cell homeostasis by maintaining the balance between synthesis and degradation, plays an important role in the pathology of different neuro-amyloidogenic diseases [8]. Further understanding of the potential role of autophagy in PD may lead to new therapeutic approaches. Thus, the present review highlights the important key players in autophagy machinery related to the pathology of PD. We also discuss the relationship between autophagy and the various risk factors for the disease.

## 2. Autophagy

Several neurological diseases are linked with neurotoxic accumulation of protein aggregates within the cells, whose degradation may be hampered, leading to cell death [8,9,10]. Autophagy is one of the main degradation and recycling processes aimed at maintaining cellular homeostasis [11]. It functions by clearing misfolded proteins and defective organelles, and by recycling cytosolic components under stress [12]. In eukaryotic cells, there are three major types of autophagy, macroautophagy, microautophagy, and chaperone-mediated autophagy (CMA), which are important for the delivery of cargo to the lysosome for degradation [13]. Hereafter, we refer to macroautophagy as autophagy. During autophagy, cytoplasmic cargo is sequestered to the autophagosome (a double-membrane vesicle), which is then fused with the lysosome for degradation. Various stimuli may lead to nonselective autophagy which in turn leads to bulk degradation of cytosolic components; autophagy can also be selective, in regard to degradation of mitochondria (mitophagy) (Figure 1) or to the clearance of specific protein aggregates [11,14]. Basal autophagy occurs in normal cells as one of the mechanisms leading to the removal of unnecessary and defective proteins and organelles. Induction of autophagy can occur under stress, in order to maintain cell homeostasis. Compared to canonical autophagy, which involves the degradation through the lysosome, it was demonstrated that autophagy-related proteins are involved in other processes called non-canonical autophagy [15]. Defective autophagy has been documented in various neurodegenerative disorders, including PD [14]. Autophagy is characterized by several sequential steps: induction and nucleation, cargo sequestration, delivery and fusion of the autophagosome with the lysosome, degradation and recycling of the degraded material, and autophagic lysosome reformation (ALR) (Figure 1) [16]. Autophagy is regulated by several independent machineries: unc-51-like autophagy-activating kinase 1 (ULK1) complex, class-III PI3K complex, and two ubiquitin-like conjugation systems [14]. The induction step involves the activity of autophagy-related (Atgs) proteins and is regulated by upstream pathways. These include the mammalian target of rapamycin complex 1 (mTORC1) pathway, which downregulates the ULK1 complex activity and is a major repressor of autophagy induction. Moreover, the Bcl-2 pathway inhibits the class-III PI3K complex by binding one of its major components, Beclin 1 [17,18,19,20]. Bcl-2 is regulated mainly by JNK1. The mTORC1 pathway is negatively regulated by energy-sensitive kinases such as AMP-activated protein kinase (AMPK) and positively regulated by Akt. The calcium signaling pathway can also modulate autophagy machineries [21]. The class-III PI3K complex is positively regulated by autophagy and Beclin 1 regulator 1 (Ambra1) and negatively regulated by UV radiation resistance-associated (UVRAG) proteins [13]. The expansion of the phagophore membrane is mediated by two ubiquitin-like conjugation systems: first, the ATG5-ATG12 connected non-covalently to ATG16L1 is associated with the phagophore membrane and is responsible for membrane elongation and autophagosome formation. The second complex involved in phagophore elongation and cargo recognition is Atg8/LC3 (light chain 3), also called MAP1LC3B (microtubule-associated protein light chain 3B), which undergoes lipidation (conjugation to phosphatidylethanolamine (PE)). The lipidation of LC3 in mammalian cells is accelerated under conditions of stress or starvation [13]. Cargo selection and sequestration processes involve the activity of adaptor proteins such as p62/SQSTM1, which recognizes ubiquitinated proteins, protein aggregates, and organelles such as damaged mitochondria. The autophagosome is then delivered to the lysosomal machinery via the microtubule network [13]. Finally, the autophagosome fuses with the lysosome, creating an autolysosome. UVRAG can activate GTPase RAB7, which promotes fusion with the lysosome. Moreover, VAM7 and VAM9 have a role in the fusion [13]. The cargo is degraded and recycled in the autolysosome.

## 3. PD Risk Factors That Affect Autophagy

Increasing evidence indicates that unregulated autophagy can contribute to the development of various neurodegenerative diseases, mainly those related to protein conformational disorders, by enhancing the accumulation of proteins and inducing cellular toxicity [12]. The effects of disrupted autophagy vary depending on the stage of the autophagic blockage and have previously been described [22,23,24,25]. Defects in initiation or cargo recognition can lead to toxicity due to accumulation of the cargo in the cytosol [26]. Impaired autophagy was evident in the brain regions of PD patients [27]. Moreover, Lewy bodies (LB) in the substantia nigra (SN) of PD brains express elevated levels of autophagy-related LC3 protein [28,29]. It was reported that in the brains of PD patients there is reduction in the levels and activities of lysosomal enzymes, such as glucocerebrosidase (GCase) or the protease cathepsin D (CTSD) [30]. Furthermore, decreased levels of the heat shock cognate 70 (HSC70) protein (also called HSPA8) and the lysosomal-associated membrane protein 2A (LAMP2A) were also found in the SN of PD cases [28,31]. The loss of CMA markers correlated with α-Syn accumulation [31]. Moreover, LB in the SN of PD brains express elevated levels of autophagy-related LC3 protein [28,29]. In the next section, we describe PD risk factors that are also involved in autophagy.

Several genes involved in PD pathology were also reported to regulate autophagy (Table 1).

**LRRK2**

Among the proteins involved in PD pathology is the leucine-rich repeat kinase 2 (LRRK2), also called dardarin or PARK8 (Table 1 [32]). It is a large, multifunctional serine–threonine kinase which also has GTPase activity. Mutations in *LRRK2* account for many autosomal dominant cases of PD. These include G2019S and R1441C mutants which are common pathogenic variants responsible for about 5% of familial PD cases [32]. Mutation in G2019S results in elevation of kinase activity [33]. Pathogenic mutations of LRRK2 severely alter its expression levels and/or kinase activity [34,35], suggesting that increased phosphorylation of LRRK2 kinase substrates may affect viability of dopaminergic neurons [36,37]. There is evidence that *LRRK2* plays a role in the regulation of autophagy. It was suggested that mutation in LRRK2 affects the maturation of autophagosomes, as shown by the reduced co-localization of LC3 with lysosome-associated membrane protein 1 (LAMP1) [38]. Fibroblasts from PD patients with *LRRK2* G2019S mutation show increased basal autophagy through activation of the MEK/ERK pathway [39]. It was also shown that LRRK 2 regulates autophagy through a calcium-dependent pathway involving nicotinic acid adenine dinucleotide phosphate (NAADP) [40] and that LRRK2 modulates lysosomal calcium homeostasis, which affects autophagy and cell survival [41]. Moreover, mutations in *LRRK2* were found to be associated with increased accumulation of phosphorylated α-Syn and increased neuronal secretion of α-Syn. Several studies suggest that LRRK2 regulates lysosomal function through its kinase activity on Rab GTPases [37]. It was also shown that mutation in LRRK2 increases the interaction between α-Syn and lysosomal membranes. LAMP2A oligomerization is inhibited by mutant LRRK2; thus, α-Syn bound to lysosomal membrane cannot translocate into the lysosome, resulting in an increased level of α-Syn oligomers in the lysosomes [37]. Overexpression of LRRK2 affects Rab phosphorylation and leads to lysosomes defects [42,43]. Depletion and inhibition of the kinase activity of LRRK2 in macrophages and microglia increased the expression of lysosomal hydrolases and enhanced autophagy [44]. Silencing of LRRK2 leads to impairment of macroautophagy and CMA [45]. Taken together, LRRK2 mutations can block CMA, stimulate autophagy, or affect the autophagy and mitophagy flux, and can influence lysosomes.

**GBA**

The glucosylceramidase beta (GBA) protein is lysosomal GCase enzyme, which maintains glycosphingolipid homeostasis. Although involved in many lysosomal storage diseases, GBA mutations are also involved in approximately 5–15% of PD patients. The common GBA mutations in PD patients are N370S and L444P [46]. Misfolded GBA impairs ER quality control by chaperone-mediated autophagy in Parkinson’s disease [47]. Loss-of-function mutations in *GBA* promote α-Syn accumulation [48]. It was also shown that in sporadic PD, GBA deficits correlate with early accumulation of α-Syn, and impairment of CMA and lipid metabolism [49]. It was also shown that GBA deficiency promotes α-Syn accumulation through inhibition of autophagy by inactivated PPP2A [50]. Furthermore, ALR is compromised in cells lacking functional GCase. In models of GCase deficiency (cells with GBA1 mutations), the levels of phospho-S6K decreased, and Rab7 GTPase, which functions in endosome–lysosome trafficking, accumulated, indicating that lysosomal recycling is impaired [51]. Moreover, GCase deficiency in neurons leads to enlargement of lysosomes and increased levels of α-Syn [52]. Interestingly, both *LRRK2* and *GBA* play a role in the autophagy-lysosomal pathway and their mutations cause similar dysfunction in autophagy and lysosomal function, resulting in aggregation and propagation of α-Syn. Indeed, neurons with *LRRK2* mutations showed reduced GCase activity [53]. LRRK2 inhibitor can restore GCase activity. Rab10, which is regulated by LRRK2, is a key mediator of GCase activity [53]. LRRK2 inhibitor reduced pS129-α-Syn levels in *LRRK2*-mutant and in *GBA*-mutant neurons [35].

**PINK1 and PRKN**

Mutations in *PINK1* and *PRKN* leading to loss function are the most common causes for early onset PD [54,55,56]. PINK1 and Parkin function in the mitochondrial quality control pathways in response to mitochondrial damage [57]. PINK1 rapidly accumulate on the outer mitochondrial membrane following mitochondrial stress and is activated by autophosphorylation thus it functions as a sensor of mitochondrial damage [57,58,59]. Following mitochondrial injury PINK1 phosphorylates both ubiquitin and the ubiquitin-like domain of Parkin, which stimulates its E3 ligase activity [59]. Parkin is an E3 ubiquitin ligase, with minimal basal activity [60]. PINK1 activates Parkin which functions as E3-Ub ligase, ubiquitinating and mediating the clearance of numerous mitochondrial proteins [61]. Mutations in PINK1 and Parkin inhibit mitophagy, thus enabling the accumulation of damaged mitochondria and possibly inducing apoptosis [62] (Table 1). One of the prominent targets of Parkin-mediated ubiquitination is mitofusins, which are GTPases essential for mitochondrial fusion [63]. It was also shown that phosphorylated ERK/MAP kinases are localized to the mitochondria and autophagosomes in LB diseases, suggesting a PD-related abnormal mitophagy [64]. Furthermore, the phosphorylated ubiquitin kinase PINK1 and the E3 ubiquitin ligase Parkin levels are increased in the PD brains and are colocalized with markers of mitochondria, autophagy, and lysosome [65].

**DJ-1**

Mutation of the *DJ-1 (PARK7)* gene causes early-onset familial PD [66,67]. DJ-1 protein is located in the cytosol and is present in the nucleus and mitochondria. Under oxidative stress, DJ-1 translocates to the mitochondria and acts as a neuroprotective intracellular redox sensor [68,69]. DJ-1-deficient mice are more sensitive to MPTP (1-methyl-4-phenyl-1,2,3,6-tetrahydropyridine)-induced loss of dopaminergic neurons [69]. It was shown that DJ-1 deficiency increases the accumulation and aggregation of α-Syn. It suppressed upregulation of LAMP2A and downregulated the level of lysosomal 70 kDa HSC70 [70]. It also regulates the inflammatory response during injury by activation of the ATG5-ATG12-ATG16L1 complex [71]. We have previously suggested that in DJ-1-deficient microglia, a gene affiliated with PD, there is an impairment in their ability to degrade and clear neurotoxic α-Syn [72]. Furthermore, we found that that DJ-1 deficiency impairs the number of lipid rafts and α-Syn uptake by microglia [72]. Under oxidative stress, increased association between DJ-1 and PINK1 were reported [67,73]. Furthermore, reduction of DJ-1 was shown to affect mitochondrial functions, its membrane potential (MMP), fusion, and fragmentation [67,69]. It was also shown that DJ-1 is essential for PINK1/Parkin-mediated mitophagy [74].

**PARK9**

Mutations in the lysosomal ATPase, ATP13A2 (PARK9), cause early-onset forms of PD. It was demonstrated that ATP13A2 functions as a lysosomal H^+^,K^+^-ATPase, contributing to acidification and α-Syn degradation in lysosomes [34]. It was reported that ATP13A2 suppresses α-Syn toxicity and its silencing can affect autophagic degradation of A53T mutant α-Syn [75,76].

**RAB39B**

RAB39B is a small GTPases important for regulation of vesicular trafficking between membrane compartments. Mutations in the *RAB39B* gene have been associated with early-onset PD [77]. Deficiency of RAB39B has been shown to impair autophagy and upregulate α-Syn in dopaminergic neurons by inducing mitochondrial dysfunction and oxidative stress [78].

**APOE4**

Several reports describe a potential connection between apolipoprotein E (APOE) isoforms and autophagy efficiency [79,80,81,82,83]. *APOE4* allele was reported to promote amyloid-beta accumulation into senile plaques in Alzheimer’s disease (AD) [84]. Other studies have suggested a connection between APOE expression and PD [85], and that APOE4 allele increases the risk of motor deficiency and decreases the age of PD onset [86,87,88]. It was suggested that PD patients carrying the *APOE3/APOE4* and *APOE4/APOE4* genotype have greater risk of dementia [89,90,91]. It was previously reported that expression of APOE4 in α-Syn transgenic mice exacerbates pathology, as shown by increased α-Syn aggregation, neuronal and synaptic loss, and impaired behavioral performances [92,93]. We have reported that in immortalized astrocytes derived from target replacement transgenic mice that express either APOE3 or APOE4, autophagy is impaired in cells expressing APOE4 [79]. We have also shown that APOE4 cells exert mitochondrial and mitophagy impairment [83].

**VPS35**

Additional proteins were implicated in Parkinson’s pathology. Vacuolar protein sorting 35 ortholog (VPS35) plays a major role in the retrograde sorting and recycling of cargo proteins. It regulates their transport from endosomes to the plasma membrane and trans-Golgi network. It was shown that VPS35 Parkinson mutation impairs autophagy via the WASH complex [94].

**WDR45**

WD repeat domain 45 protein (WDR45) plays a role in cell cycle control, translational regulation, signal transduction, and autophagy [95]. WDR45 mutation impairs the autophagic degradation of transferrin receptor and promotes ferroptosis [96,97]. Patients with a WDR45 defect have neurodevelopmental disorder and late-onset Parkinsonism [98].

**FBXO7**

Reduced FBXO7 (F-box-only protein 7) expression results in deficiencies in mitochondrial Parkin, ubiquitination of mitofusin 1, and mitophagy [99]. Inhibition of FBXO7 reduces inflammation and induces neuroprotection by stabilizing PINK1 [100].

## 4. The Role of Autophagy Impairment and α-Syn Pathology in PD

The main pathologies in PD include the accumulation of LB and the death of dopaminergic neurons in the substantia nigra (SN), leading to reduction of dopamine production in the brain. LB, containing α-Syn aggregates, serve as a marker for α-synucleinopathies typical for PD, and are frequently found in the SN, and during the disease progression, they are more diffused throughout the brain [2]. There are two main theories regarding the loss of the dopaminergic neurons in the SN. The first is related to α-Syn aggregates which are commonly observed in PD patients, and the second suggests damage due to mitochondrial dysfunction [67]. α-Syn is part of the synuclein family of proteins: α-synuclein, β-synuclein, and γ-synuclein. β-synuclein and γ-synuclein were implicated in human brain lesions and were found to be co-expressed with α-Syn in LB [101,102]. α-Syn (14KDa) is mainly expressed in the brain at the pre-synaptic terminals, associated with vesicular release, although its exact function is still unclear. It can also be found in the cerebrospinal fluid (CSF), blood, and plasma [2].

Spreading of α-Syn within the dopaminergic neuronal cells was suggested as one of the processes affiliated with the progression of the disease, by inducing neurotoxicity of the recipient cells [103]. It is secreted following stress, lysosomal dysfunction, aggregation, inhibition of the proteasome and mitochondrial dysfunction, and the release from the cell also depends on its specific conformation [103]. α-Syn production, aggregation inside cells, uptake by neighboring cells, and degradation rate either inside or outside cells will determine the extent of affected neurons [104]. The protein is transmitted by endocytosis, plasma membrane penetration, or exosomes [103]. α-Syn uptake can also occur by diffusion, by endocytosis or phagocytosis. Several mechanisms for α-Syn uptake were suggested [105]. It was suggested that lymphocyte activation gene 3 (LAG3) is the receptor with a high affinity to α-Syn [104,106]. However, another study did not find expression of LAG3 in brain neurons, and overexpression or depletion of LAG3 had no effect on α-Syn pathology in A53T mice [107]. Notably, toll-like receptors (TLRs), especially TLR2 and TLR4, are dysregulated in patients with PD. A connection between these TLRs’ expression and α-Syn aggregation was suggested [108]. In addition, α-Syn aggregation was previously suggested to activate NLRP3-type inflammasome (nucleotide-binding oligomerization domain-leucine-rich repeat-pyrin domain-containing 3) [109]. NLRP3 was reported to play a role in mediating neuroinflammation and autophagy in PD [110,111,112].

Several studies indicate that α-Syn can affect autophagy. It was reported that overexpression of α-Syn increases the interaction between Bcl2 and BECN1, thus inhibiting autophagy [113]. Moreover, α-Syn inhibits microglia autophagy [114]. It was demonstrated that α-Syn leads to the accumulation of Parkin (PRKN) [115] and that it compromises autophagy via inhibition of Rab1, resulting in ATG9 mislocalization [116]. It was also shown that expression of α-Syn impairs autophagolysosome maturation [117] and decreases the abundance of SNAP29, a member of the SNARE complex that mediates autophagolysosome fusion [118]. In addition, overexpression of α-Syn showed disruption of lysosomal morphology and distribution [119]. Interestingly, it was found that autophagic receptor protein SQSTM1/p62 S nitrosylation inhibits autophagic flux and promotes the accumulation of misfolded α-Syn. This modification increases the secretion and spread of aggregated synuclein, thus contributing to autophagy inhibition, neuronal damage, and the propagation of α-Syn in the brain [120].

Degradation of α-Syn aggregates depends on autophagy-mediated lysosomal degradation [121]. Generally, monomers are degraded by CMA [106,121], while autophagy has an important role in aggregate degradation [12], although the degradation mechanism also depends on the posttranslational modification of the proteins [121,122]. Nevertheless, α-Syn mutations, phosphorylation, ubiquitination, nitration, oxidated forms, and posttranslational modified forms induced by dopamine have been identified in cytosolic aggregates in brains of PD patients and in experimental models of the disease, and have been shown to inhibit autophagy [122,123,124,125]. Several genetic mutations in ATG genes may lead to different phenotypes of PD [8]. In addition, elevated levels of intracellular α-Syn due to inhibition of the GTPase Rab1A have been shown to hamper omegasome formation by affecting Atg9 localization [116].

Since hyperphosphorylated α-Syn has propagational properties, it was suggested that its level decreases during autophagy. Klucken et al. have shown evidence for the function of the autophagy-lysosomal pathway (ALP) in α-Syn degradation. Inhibition of ALP by bafilomycin A1 (BafA1) enhanced the toxicity of aggregated α-Syn; however, reduced toxicity correlated with α-Syn aggregation, suggesting that protein aggregation may be a detoxification event [126]. Another study has shown that ALP inhibition by BafA1 reduced intracellular α-Syn aggregation and increased the secretion of smaller oligomers. This effect worsened cell responses including uptake, inflammation, and cellular damage. Low-aggregated α-Syn release was mediated by exosomes and RAB11A-associated pathways; however, high-aggregated α-Syn was mediated by membrane shedding [127]. This study suggests that impaired ALP limits intracellular degradation of misfolded proteins, and also leads to increased α-Syn secretion and propagation in the brain [127]. Autophagy was also considered in several papers to play an important role in glial cells’ ability to clear neurotoxic α-Syn [128]. Astrocytes were reported to endocytose extracellular α-Syn released by neurons and play a role in its spreading [129,130]. It was previously suggested that autophagy plays an important role in the ability of astrocytes to clear and degrade neurotoxic components [131]. Furthermore, according to Tsunemi et al., astrocytes have a higher proteolytic capacity of α-Syn than neurons, and co-culturing of astrocytes and neurons decreases the transfer of α-Syn between neurons [130].

## 5. Targeting Autophagy as Therapeutic Approach in PD

Several autophagy-enhancing agents were tested in pre-clinical PD models [132,133]. These include drugs that affect the mTOR signaling pathway such as rapamycin, metformin, resveratrol, PREP inhibitor (KYP-2047), and isorhynchophylline. Other drugs that affect the mTOR independent pathway include Lithium, Sodium Valproate, Carbamazepine, Trehalose, Latrepirdine, Spermidine, and Nilotinib [132,133]. Another approach is to use drugs that affect lysosome function such as Ambroxol, Isofagomine, and acidic nanoparticles. These drugs can also affect cell viability, the levels of phosphorylated α-Syn, the rate of α-Syn clearance, the mitochondrial function, lysosomal functions, and neuroinflammation [132,133]. Several studies demonstrated that the symptoms induced by MPTP (1-methyl-4-phenyl-1,2,3,6-tetrahydropyridine) in mice are improved following treatment with rapamycin [134,135]. Rapamycin and trehalose treatments have been shown to exert protective effect on damaged dopaminergic neurons (induced by MPTP) [136]. Additional autophagy-targeting compounds that can be used as potential treatment have been described [137]. It was demonstrated that in models of PD in vivo and in vitro, autophagy inhibition at various stages has positive effects on reduction of α-Syn levels of inclusion; it increased α-Syn clearance, cell viability, and protection from α-Syn toxicity [137].

Another approach to treating PD pathology was suggested by targeting mitophagy. Targeting PINK1/Parkin dependent and independent pathways has been suggested. For example, PINK1 activator KTP (kinetin triphosphate) and its analogues and niclosamide and its analogues have been described [138,139,140,141].

LRRK2 is considered as a good target to develop drugs for PD treatment [142]. It has been shown that LRRK2 kinase inhibitor, GSK3357679A, can rescue LRRK2 G2019S knock-in mice from their defects in mitophagy [143]. Recently, small-molecule LRRK2 inhibitors for PD therapy were described with the potential that they will prove efficient for PD treatment [141]. Moreover, new ongoing clinical trials to evaluate the efficiency of LRRK2 inhibitors, BIIB122 and DNL-201, for treatment of PD patients have been conducted [144,145,146].

Mutations in the ATP13A2 gene, which appears in PD patients, have been described previously. Indeed, it was shown that drugs such as clioquinol and rifampicin restore lysosomal acidification in mammalian models of PD [147]. Moreover, the compound ML-SA1, which increases lysosomal function, has been shown to reduce SNCA accumulation in DA neurons of PD patients [133].

Another approach to modulating autophagy as treatments for PD are miRNAs. A neuroprotective role of agents that affect autophagy-regulating miRNAs has been described [148]. Agents such as Baicalein and Pramipexole have been reported to alleviate PD characteristics [148].

Further studies are needed to determine the benefit of a therapeutic approach that enhances autophagy/mitophagy functions for treatment of PD patients. However, it is extremely important to understand the molecular mechanisms in neuronal and non-neuronal cells and to assess the most potent genes and pathways involved as targets for therapeutic application.

## 6. Concluding Remarks

PD is neuropathologically characterized by progressive loss of dopaminergic neurons, accompanied by accumulation of LB with α-Syn aggregates as the major protein component [36]. Degradation pathway dysfunctions, such as autophagy and lysosome, were also described [29,149]. Autophagy, as one of the major degradation pathways, plays a pivotal role in maintaining effective protein and organelle turnover, maintaining cell homeostasis and preventing toxicity and cell death. Accumulating evidence suggests that increased α-Syn aggregates are a consequence of impaired autophagy [121]. In turn, α-Syn was also shown to affect mitochondrial, autophagic, and lysosomal functions [121,150,151,152]. Moreover, several mutations in genes linked to PD were implicated in early-onset familial PD [36]. Recent studies, including our own, suggest that other factors, such as APOE4 expression, which are involved in other neurodegenerative diseases, may affect autophagy/mitophagy processes and possibly can be linked to PD as well [82,153]. Taken together, these findings may suggest a pivotal role of autophagy within the pathology of PD. Further research on autophagy pathways may increase our understanding of the etiology of the disease towards development of future therapeutic intervention.

**Table 1:** Proteins involved in PD that affect autophagy.

Protein	Connection to PD	Autophagy/Mitophagy/CMA	Bibliography
α-synuclein	Mutations in α-synuclein are affiliated with early PD onset and Lewy bodies formation.	Inhibits autophagy in the nucleation and maturation steps. Inhibits mitophagy.	[31,103,104,105,106,107,109,110,111,112,113,114,115,116,117,118,119]
DJ-1	Deletions and point mutations of DJ-1 cause autosomal recessive PD.	Regulates autophagy/mitophagy.	[66,67,68,69,70,71,72,73,74]
LRRK2	*LRRK2* mutations are found in families with late-onset autosomal-dominant PD.	Affects autophagy, mitophagy, and CMA.	[27,32,33,34,35,36,37,38,39,40,41,42,43,44,45]
Apolipoprotein E4	APOE4 isoform was suggested to be involved in PD.	Impairs autophagy and mitophagy.	[79,80,81,82,84,85,86,87,88,89,90,91,92,93,154]
PTEN-induced kinase 1 (PINK1) and PARKIN	Loss of function mutations of PINK1 and PARKIN genes are the most common causes of autosomal recessive and early-onset PD.	Regulate mitophagy.	[54,55,56,57,58,59,60,61,62,63]
GBA	Mutations in the *GBA* gene lead to lysosomal dysfunction and impaired α-synuclein metabolism.	Affects lysosomal activity.	[46,47,48,49,50,51,52,53]
VPS35	Mutations in the *vacuolar protein sorting 35 ortholog* (*VPS35*) gene cause late-onset autosomal dominant familial PD.	Inhibits autophagy in the nucleation step.	[94]
RAB39B	*RAB39B* gene mutations were associated with X-linked neurodevelopmental defects including early-onset Parkinson’s disease (PD).	Affects autophagy/mitophagy.	[77,78,155,156]
ATP13A2 (PARK9).	*ATP13A2* (*PARK9*) gene mutations cause Kufor–Rakeb syndrome (Parkinson’s disease 9), an autosomal recessive form of Parkinsonism with dementia.	Affects lysosome activity and α-syn degradation.	[34,75,76,157,158,159,160]
WDR45	WDR45 (WD Repeat Domain 45) is a component of the autophagy machinery that controls the major intracellular degradation process.	Regulates autophagy.	[95,96,97,98]
FBXO7	*FBXO7* (F-box-only protein 7) *gene* mutations have been identified in a number of families with severe autosomal recessive early-onset Parkinson’s disease.	Affects mitophagy.	[99,100]

## Abbreviations

α-Syn	α-synuclein
MPTP	1-methyl-4-phenyl-1,2,3,6-tetrahydropyridine
Ambra1	Activating molecule in Beclin1-regulated autophagy
AD	Alzheimer’s disease
AMPK	AMP-activated protein kinase
APOE	Apolipoprotein E
ALP	Autophagy-lysosomal pathway
ALR	Autophagic lysosome reformation
Atgs	Autophagy-related proteins
AV	Autophagic vacuoles
BafA1	Bafilomycin A1
CSF	Cerebrospinal fluid
CMA	Chaperone-mediated autophagy
CTSD	Protease cathepsin D
FBXO7	F-box-only protein 7
GCase	Glucocerebrosidase
GBA	Glucosylceramidase beta
HSC70	Heat shock cognate 70
LRRK2	Leucine-rich repeat kinase 2
LB	Lewy bodies
LC3	Light chain 3
LAG3	Lymphocyte activation gene 3
LAMP	Lysosome-associated membrane protein
mTORC1	Mammalian target of rapamycin complex 1
MMP	Mitochondrial Membrane Potential
NAADP	Nicotinic acid adenine dinucleotide phosphate
NLRP3	Nucleotide-binding oligomerization domain-leucine-rich repeat-pyrin domain-containing 3
PD	Parkinson’s disease
PE	Phosphatidylethanolamine
PINK1	PTEN-induced kinase 1
SN	Substantia Nigra
SNCA	Synuclein alpha
TLRs	Toll-like receptors
ULK1	Unc-51-like autophagy-activating kinase 1
VPS35	Vacuolar protein sorting 35 ortholog
WDR45	WD Repeat Domain 45
